# Charge transport in a zinc–porphyrin single-molecule junction

**DOI:** 10.3762/bjnano.2.77

**Published:** 2011-10-18

**Authors:** Mickael L Perrin, Christian A Martin, Ferry Prins, Ahson J Shaikh, Rienk Eelkema, Jan H van Esch, Jan M van Ruitenbeek, Herre S J van der Zant, Diana Dulić

**Affiliations:** 1 Kavli Institute of Nanoscience, Delft University of Technology, Lorentzweg 1, Delft, The Netherlands; 2 Department of Chemical Engineering, Delft University of Technology, Julianalaan 136, 2628 BL Delft, The Netherlands; 3 Kamerlingh Onnes Laboratory, Leiden University, Niels Bohrweg 2, 2333 CA Leiden, The Netherlands

**Keywords:** mechanically controllable break junction, molecular conformation, molecular electronics, porphyrin, single-molecule transport

## Abstract

We have investigated charge transport in ZnTPPdT–Pyr (TPPdT: 5,15-di(*p*-thiolphenyl)-10,20-di(*p*-tolyl)porphyrin) molecular junctions using the lithographic mechanically controllable break-junction (MCBJ) technique at room temperature and cryogenic temperature (6 K). We combined low-bias statistical measurements with spectroscopy of the molecular levels in the form of *I*(*V*) characteristics. This combination allows us to characterize the transport in a molecular junction in detail. This complex molecule can form different junction configurations, having an observable effect on the trace histograms and the current–voltage (*I*(*V*)) measurements. Both methods show that multiple, stable single-molecule junction configurations can be obtained by modulating the interelectrode distance. In addition we demonstrate that different ZnTPPdT–Pyr junction configurations can lead to completely different spectroscopic features with the same conductance values. We show that statistical low-bias conductance measurements should be interpreted with care, and that the combination with *I*(*V*) spectroscopy represents an essential tool for a more detailed characterization of the charge transport in a single molecule.

## Introduction

The break-junction method represents a popular choice to investigate the electronic transport through metal–molecule–metal junctions [1–6]. While repeatedly breaking and fusing two metallic electrodes, the low-bias conductance is monitored as a function of the electrode displacement. Such low-bias transport measurements have been extensively used to study the dependence of the molecular conductance on the length [1–2], conformation [3–4] and anchoring groups [5–6] of rod-like molecules. However, as the bias range is very limited, the main contribution to the current is off-resonance transport. As such, spectroscopic information about molecular energy levels involved in the charge transport is lacking.

Here, we investigate charge transport through a zinc(II) porphyrin [zinc(II) 5,15-di(*p*-thiolphenyl)-10,20-di(*p*-tolyl)porphyrin] with an axial pyridine ligand in both the low-bias and the high-bias regime. Porphyrins are interesting for this purpose as they are complex, non-rodlike molecules, which can form different stable conformations [7–8], especially when functionalized with metal-bound axial pyridine ligands [9]. Using the mechanically controllable break-junction (MCBJ) technique, we study the low-bias conductance as a function of the electrode displacement. In addition, we perform current–voltage measurements at different electrode spacings in order to gain spectroscopic information in the high-bias regime.

**Figure 1:**
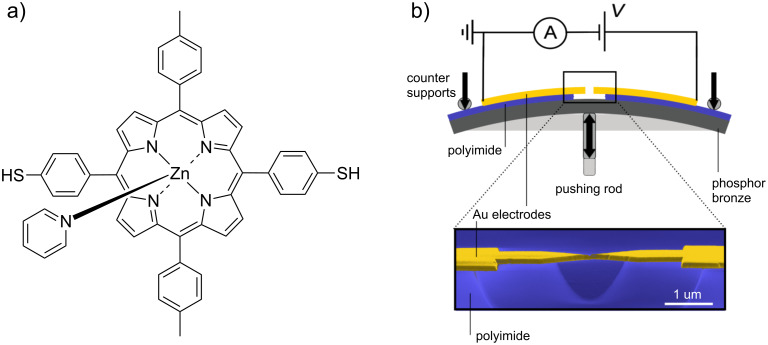
Structural formula of ZnTPPdT–Pyr (b) Top: Setup of the mechanically controllable break-junction (MCBJ). Bottom: Scanning electron micrograph of a MCBJ device (colorized for clarity). The scale bar shows that the suspended bridge is about 1 µm in length.

The MCBJ technique is an elegant way to control the spacing between two metallic electrodes with subatomic (<10^−10^ m) resolution [10–12]. This control is achieved by bending a substrate supporting a pair of partially suspended electrodes, in a three-point bending mechanism. Upon bending of the substrate, a nanosized gap is formed between the electrodes, which can be mechanically adjusted and which is impressively stable on the order of several hours, even at room temperature [13–14]. The layout of the technique is schematically presented in Figure 1b.

All experiments were performed in high vacuum (<10^−6^ mbar). Prior to the experiments, a complex of zinc(II) 5,15-di(*p*-thiolphenyl)-10,20-di(*p*-tolyl)porphyrin and pyridine (ZnTPPdT–Pyr) (see Figure 1a for the structural formula) was dissolved in dichloromethane (DCM) and deposited on the unbroken electrodes by means of self-assembly from solution. Two thiol groups on opposite sides of the molecule are used as anchoring groups. After deposition, the junctions are broken in vacuum at room temperature. The aforementioned stability of the electrodes allows us to characterize charge transport through ZnTPPdT–Pyr by performing two types of experiments. First, we measure at room temperature the low-bias conductance of the molecule as a function of the electrode stretching. Second, we perform spectroscopy of the molecular energy levels by measuring current–voltage characteristics at fixed electrode spacings; this was done both at room temperature and cryogenic temperature (6 K).

## Results

To obtain the conductance value of the most probable contact geometry we repeatedly broke and fused the electrodes [15–17] between conductances of 1·10^−5^* G*_0_ and 10 *G*_0_, while measuring the current at a fixed bias voltage (100 mV). Each breaking event produced a “breaking trace” of the conductance, which is plotted as log_10_(*G*) versus the electrode displacement *d*. Sets of 500 consecutive breaking traces from individual junctions were then binned in time and in electrode displacement. As we are interested in the breaking dynamics of the junctions beyond the point of rupture of the last monatomic gold contact (defined as *d* = 0), only conductance values below one quantum unit *G*_0_ = 2e^2^/h (the resistance of a single gold atom) are considered. The results are plotted as two-dimensional “trace histograms”, in which areas of high counts represent the most typical breaking behavior of the molecular junction [18–19].

**Figure 2:**
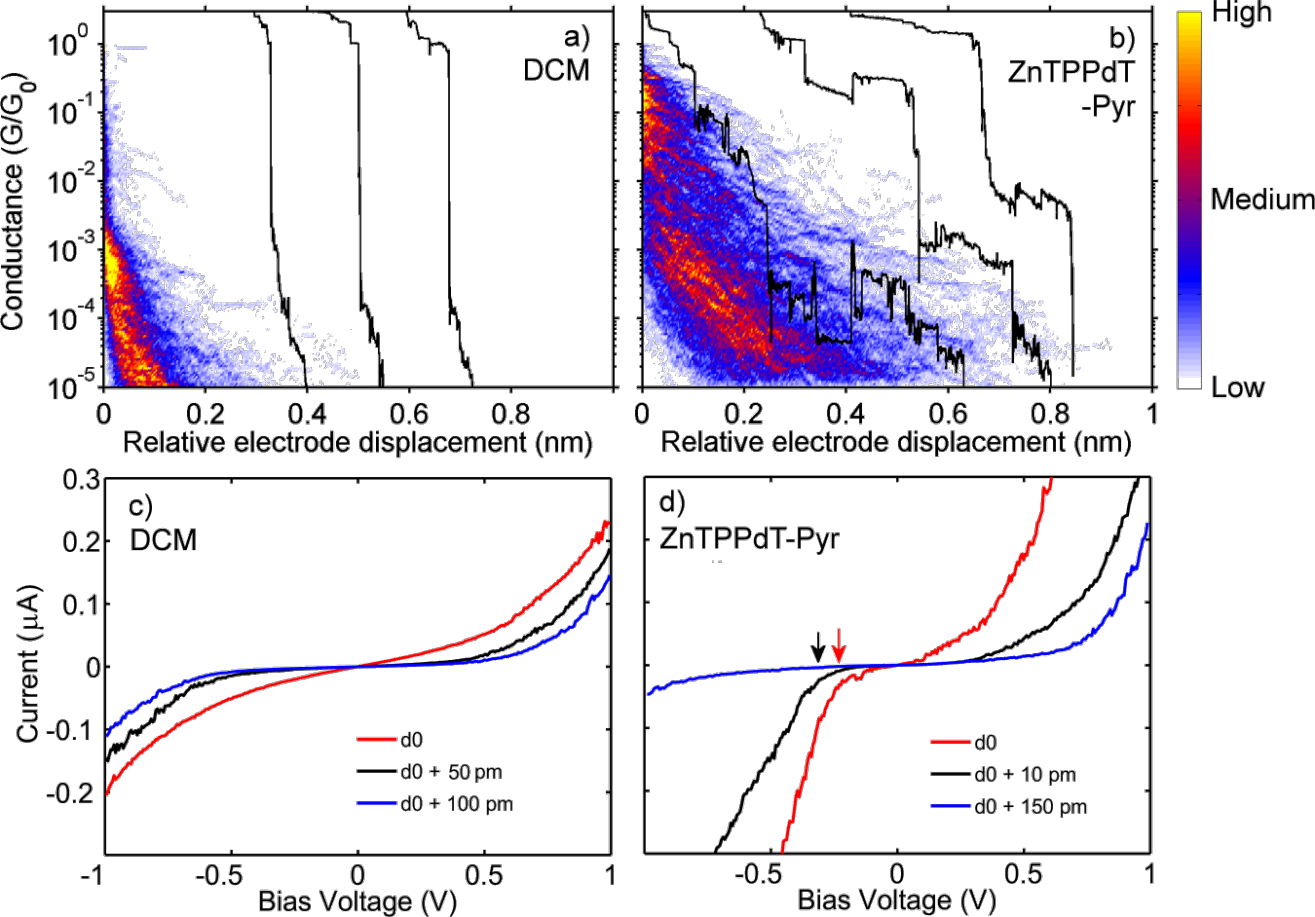
Trace histograms constructed from 500 consecutive breaking traces taken at room temperature and 100 mV bias for junctions exposed to (a) the solvent DCM only, and (b) to ZnTPPdT–Pyr. Regions of high counts represent the most probable breaking behavior of the contact. The black curves are examples of individual breaking traces (offset along the horizontal axis, *d*, for clarity). For the construction of the trace histograms, the zero of the relative electrode displacement for each curve was set to the point where the conductance drops sharply below 1 *G*_0_. (c) Current–voltage characteristics taken at various electrode spacings starting from the initial value *d*_0_ of junctions exposed to the solvent DCM, and (d) to ZnTPPdT–Pyr.

In Figure 2, we show trace histograms as well as examples of individual breaking traces for a junction exposed to (a) the solvent DCM and (b) ZnTPPdT–Pyr. All measured curves are included, i.e., no data selection was employed. We measured several samples with ZnTPPdT–Pyr molecules as well as DCM references. The features shown in Figure 2a and Figure 2b are representative of all these measurements. In the junction that was exposed to the pure solvent without porphyrin molecules (Figure 2a), the Au-bridge is stretched until a single-atom contact is formed, visible (only in the individual offset traces) as a plateau around the conductance quantum (*G* ~ *G*_0_). Upon further stretching, the monatomic contact is broken and the conductance decreases sharply and abruptly to ~10^−3^* G*_0_ due to relaxation of the electrode tips. Beyond this point, electron tunneling between the electrodes leads to a fast conductance decay with stretching (visible as the orange tail), as expected for tunneling through a single barrier.

In contrast to this fast tunneling decay, introduction of the porphyrin molecules by self-assembly in the junction led to pronounced plateaus at different conductance values in the sub-*G*_0_ regime. The observation of such plateaus in the breaking traces is commonly taken as a signature of the formation of a molecular junction [15–17]. Figure 2b shows that the plateaus can be horizontal or sloped. Some traces consist of a few plateaus at different conductance values. The representative breaking traces that are included in Figure 2b display a set of such plateaus. In strong contrast to measurements on rod-like molecules, averaging over 500 traces does not lead to a narrow region of high counts in the trace histograms. Instead, two distinct regions with high counts are visible; a high-conductance region around 10^−1^* G*_0_, and a sloped low-conductance region ranging from 10^−3^ *G*_0_ to 10^−5^* G*_0._ Although clear plateaus are observed in the single breaking traces, averaging over hundreds of traces washes out the molecular signature. Hence, a complementary method is required to study charge transport in more detail.

We therefore measured current–voltage characteristics (*I*(*V*)s) at a fixed electrode spacing, in the 10^−2^–10^−5^ *G*_0_ conductance region. In between the *I*(*V*) measurements, the interelectrode distance was gradually increased or decreased in steps of about 10 pm, without fusing the electrodes to form a metallic contact. In this way, changes in the configurations of the molecular junctions occurring as a function of electrode spacing can be accurately probed. *I*(*V*)s taken at room temperature for several electrode spacings of the junctions exposed to DCM and ZnTPPdT–Pyr are presented in Figure 2c and Figure 2d, respectively. For each series, all the presented *I*(*V*)s are taken from the same breaking sequence.

*I*(*V*)s of a junction exposed to DCM (Figure 2c) exhibit the characteristic single-barrier tunneling shape and show the expected current decrease upon increasing the electrode spacing. In contrast, *I*(*V*) characteristics on the ZnTPPdT–Pyr junction show a sharper current onset, marked by arrows in Figure 2d. This observation may be viewed as a molecular fingerprint as the marked points correspond to the onset of resonant transport through an energy level of the molecule (either vibrational or electronic). Interestingly, the current onset strongly depends on the interelectrode distance. At *d*_0_ it is located around −250 mV. After a step of about 10 pm in the electrode distance, the onset shifted to around −350 mV. Increasing the inter-electrode distance by an additional 140 pm, shifted the onset at negative bias to a location outside the bias window. Note furthermore the asymmetry in the curves in Figure 2d, which increases as the electrodes move further apart (i.e., the blue curve in Figure 2d). For the three *I*(*V*)s we also determined the conductance at the same bias voltage as used to construct the trace histograms, i.e., at 100 mV. For the red, black and blue *I*(*V*) curve we obtain conductance values of 2.0∙10^−3^, 1.6∙10^−4^ and 1.6∙10^−4^ *G*_0_ respectively. Interestingly, small changes in electrode distance (~10 pm) can induce significant changes in the shape of the *I*(*V*) characteristics and the low-bias conductance (compare, e.g., the red and black curves). Opening the junction further (black and blue curves) results in no change of the conductance value at 100 mV, but in different *I*(*V*) shapes.

**Figure 3:**
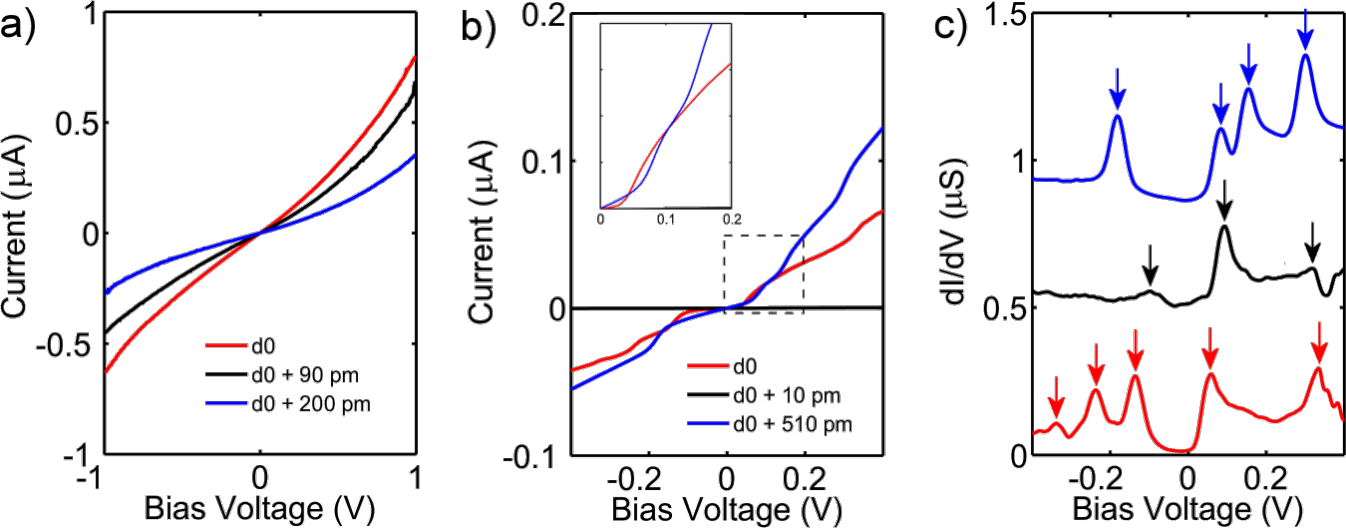
Low-temperature *I*(*V*) characteristics of junctions exposed to (a) DCM and (b) ZnTPPdT–Pyr. The DCM sample clearly shows vacuum-tunneling behavior. The porphyrin sample exhibits Coulomb blockade and steps. (c) d*I*/d*V* of a junction exposed to a ZnTPPdT–Pyr solution; curves are offset vertically for clarity. Resonances correspond to electronic or vibrational energy levels of the molecular junction. Note, for the black line the d*I*/d*V* has been scaled by a factor of 100.

Spectroscopic features become more pronounced at low temperature as the junction stability increases, and both the thermal noise and thermal broadening decrease. We therefore cooled down the junctions to cryogenic temperature (6 K) while keeping the zero-bias conductance at a fixed value (around 1∙10^−4^ *G*_0_) with a feedback loop. In Figure 3a and Figure 3b, we present low-temperature *I*(*V*)s of junctions exposed to (a) DCM and (b) ZnTPPdT–Pyr solution, for different electrode spacings. *I*(*V*)s of the junction exposed to DCM show the characteristic tunneling shape, without any molecular signature, as was also found at room temperature. A notable difference, however, is the significant reduction of the noise.

The *I*(*V*)s of the junction containing ZnTPPdT–Pyr now show sharp step-like features, which are more pronounced than those in Figure 2d. We numerically determined the differential conductance (d*I*/d*V*) as displayed in Figure 3c. In the d*I*/d*V* curves, the steplike features are visible as resonance peaks, which are marked in the figure with arrows of the corresponding color. For clarity, the d*I*/d*V* curves are offset vertically, and the d*I*/d*V*-curve represented by the black curve is magnified 100 times. The origin of these resonances can be electronic or vibrational [20–22]. Independent of their origin, their position reveals the alignment of the corresponding energy level with respect to the Fermi energy of the electrodes [23]. For a distance of *d*_0_ (red curve), five pronounced resonances are present, located at −339 mV, −283 mV, −153 mV, 58 mV and 334 mV. For the conductance at 100 mV we obtain a value of 2.1∙10^−3^ *G*_0_. Increasing the distance by 10 pm (black curve) drastically changes the molecular energy spectrum, with one distinct resonance at 94 mV, and two fainter peaks around −99 mV and 319 mV. Here, the conductance at 100 mV is 1.2∙10^−5^ *G*_0_. Increasing the distance by an additional 500 pm (blue curve) again leads to changes in the molecular energy spectrum; in this case four pronounced resonances are located at −238 mV, −136 mV, 58 mV and 334 mV. For the conductance we again obtain a value of 2.1∙10^−3^ *G*_0_.

## Discussion

Comparing first the red and black curve in Figure 3c, we see that within a change in the electrode displacement of 10 pm, the number of energy levels involved in the electronic transport as well as their exact energy drastically changed. A major jump of two orders of magnitude in the low-bias conductance was observed as well. This suggests an abrupt change in the molecule–electrode interaction, presumably caused by a change in molecular conformation. A similar change in molecular conformation was also observed in the room temperature *I*(*V*)s as demonstrated by the red and black curves in Figure 2; the onset for the current increase shifted by −100 mV and the conductance dropped by one order of magnitude within 10 pm. These observations support the conclusion drawn from the trace histogram measurements: The molecule can adopt different stable conformations, leading to plateaus at different conductance values in the breaking traces. Comparing the red and blue curves in Figure 3c, which were taken are at a separation of 510 pm, we see that their molecular energy spectra strongly differ, but that their low-bias conductance is similar (Figure 3b, inset). Similar behavior was also observed at room temperature (Figure 2d). This suggests that different stable junction configurations with very different spectroscopic signatures can exhibit the same low-bias conductance.

For most of the low-bias break-junction measurements on rod-like molecules it is assumed that repetitive fusing and breaking of the molecular junction provides the most probable conductance value [15–17]. Multiple conductance peaks are often attributed to the formation of multiple molecular bridges connected in parallel [15,24]. The strength of the molecule–metal chemical bond is considered to play a central role in determining the single-molecule conductance values. Our results on the Zn-porphyrin molecule with a pyridine axial group show that different conductance values can also result from the stretching or fusing of a molecular junction. As considerable changes in the conductance values and spectra already occur for a displacement as small as 10 pm, we conclude that neither the molecule–electrode chemical bond nor the electrode configuration itself can be held responsible. More likely, varying the electrode distance changes the molecular conformation, which in turn leads to abrupt changes in the molecule–electrode interaction. Our findings also show that *I*(*V*) characteristics taken at different electrode spacings can exhibit distinct different spectroscopic features but a similar low-bias conductance. This indicates that different junction geometries can lead to similar conductance values in the trace histograms. Therefore, as changes in the configuration of the molecular junction are not always reflected in the low-bias trace histograms, supporting high-bias *I*(*V*) characteristics are essential for the interpretation of such histograms.

## Conclusion

In summary, we investigated charge transport in ZnTPPdT–Pyr molecular junctions using the lithographic MCBJ technique. We combined low-bias statistical measurements with spectroscopy measurements of the molecular levels in the form of *I*(*V*) characteristics. This unique combination allows us to probe different junction configurations and monitor changes in the molecular-level alignment upon fusing or breaking of a molecular junction. Both methods show that multiple stable single-molecule junction configurations can be obtained by stretching or fusing the junction. In addition we demonstrate that different ZnTPPdT–Pyr junction configurations can lead to different spectroscopic features for similar low-bias conductance values. Thus, *I*(*V*)-spectroscopy measurements can provide additional information compared to statistical low-bias conductance histograms, enabling a more in-depth characterization of the charge transport through a single molecule.

## Supporting Information

Supporting Information features detailed information on sample preparations and measurement procedures.

File 1 Experimental details.
