# Supplementary material for: Charge transport in a zinc–porphyrin single-molecule junction
Source: Beilstein J Nanotechnol. 2011 Oct 18;2:714–9. doi: 10.3762/bjnano.2.77 (PMC3201625; doi:10.3762/bjnano.2.77)
Supplement: File 1 — Experimental details. [file Beilstein_J_Nanotechnol-02-714-s001.pdf]

Supporting Information

for

Charge transport in a zinc–porphyrin single-molecule junction

Mickael L. Perrin*[1], Christian A. Martin[1], Ferry Prins[1], Ahson J. Shaikh[2], Rienk Eelkema[2], Jan H. van Esch[2], Jan M. van Ruitenbeek[3], Herre S. J. van der Zant[1], Diana Dulić[1]

Address: [1]Kavli Institute of Nanoscience, Delft University of Technology, Lorentzweg 1, Delft, The Netherlands; [2]Department of Chemical Engineering, Delft University of Technology, Julianalaan 136, 2628 BL Delft, The Netherlands; and [3]Kamerlingh Onnes Laboratory, Leiden University, Niels Bohrweg 2, 2333 CA Leiden, The Netherlands

Email: Mickael L. Perrin - m.l.perrin@tudelft.nl

* Corresponding author

Experimental details

Contents

I) Synthesis of ZnTPPdT-Pyr ……………………………………………………….S2

II) Sample fabrication………………………..…………………………………………S2

III) Experimental procedures……………………………………………………………S2

IV) Calibration of the attenuation factor……..…………………………………………S3

I) Synthesis of ZnTPPdT

The synthesis of the starting material TPPdT is reported elsewhere [1]. ZnTPPdT was prepared by adapting a reported procedure [2]. A solution of $Zn(OAc)_2 \cdot 2H_2O$ (10 mg, 45.5 μmol) in MeOH (2.5 mL) was added to a solution of the free-base TPPdT (5 mg, 7.0 μmol) in THF (7.5 mL). The mixture was stirred at room temperature for 2 hours, after which TLC confirmed the completion of the reaction. The solvent was evaporated, and the product was dissolved in dichloromethane, and excess zinc acetate was removed by washing twice with water. Removal of the solvent and subsequent column chromatography yielded the product ZnTPPdT (5.4 mg, 7.0 μmol, quantitative yield). ^1H NMR confirmed the absence of pyrrolic-NH protons. MS (ESI^-) m/z $\{M-H+CF_3COO+2DMSO+CH_3CN\}^{2-}$ calcd for $C_{54}H_{48}F_3N_5O_4S_4Zn$ 538.6, obsd 538.7.

II) Sample fabrication and molecule deposition

Details concerning the sample fabrication were published in a previous paper [3]. For the molecule deposition, ZnTPPdT was dissolved in dichloromethane at a concentration of 0.1 mM. ZnTPPdT–Pyr was then made in situ during self-assembly of the porphyrin on the MCBJ, by addition of pyridine (0.1%) to the ZnTPPdT solution. Devices were submerged in the molecular solution for 1 h. After self-assembly, the devices were rinsed in pure solvent.

III) Experimental procedures

A home-made LabVIEW program was used to control the bending of the device, the measurement electronics and the data acquisition. Before each measurement, the setup was pumped down to a pressure below 10^{-6} mbar. Conductance traces were acquired at a DC bias of 100 mV. All electrical measurements were carried out with home-built, battery-operated equipment. We measured the junction currents using a logarithmic current–voltage converter with temperature-drift compensation, covering a range from less than 50 pA to more than 1 mA. Pointwise calibration with standard resistors ranging from 100 Ω to 1 GΩ ensured the accuracy of this conversion. The

junction conductance was calculated from the measured current and the applied bias voltage. All current and voltage data were read into the PC by means of an Adwin-Gold data acquisition module (Jaeger).

During the histogram measurements the flexible MCBJ substrates were bent at a speed of 2 μm/s by means of a differential screw and a PC-controlled servo motor (Faulhaber). On the scale of the electrodes, this bending is translated to a stretching on the order of 0.12 nm/s (for the calibration of the attenuation factor, see section *Calibration of the displacement ratio*). In order to acquire thousands of breaking curves the junctions were cycled repeatedly between the fused (10 G_0) and the open state ($1 \cdot 10^{-5}$ G_0). Complete opening of the contacts was guaranteed by an additional bending of 5 μm after the conductance had crossed the lower boundary of the studied conductance interval. Fusing to 10 G_0 ensured the atomic reconfiguration of the contact after each cycle.

We collected all conductance traces consecutively on individual junctions. Raw data were evaluated statistically without any data selection to obtain the trace histograms. The trace histograms were created by following the procedure described in a previous publication [3]. On the y-axis we used logarithmic binning at 30 bins per decade of conductance. On the x-axis, we used 10 bins per μm. In order to compare different measurements, the counts in the histograms were divided by the number of traces, the data acquisition rate (in Hz) and multiplied by the bending speed (in μm/s). The resulting trace histograms were plotted using a color scale with a minimum of 0 and a maximum of $4 \cdot 10^{-3}$ counts. Data analysis and plotting of the histograms was carried out with a MATLAB package (The MathWorks™).

IV) Calibration of the displacement ratio

In the metallic regime, the signature of atomic chains can be used to calibrate the displacement ratio of the MCBJ. Similar to a single-atom contact, chains of gold atoms exhibit a conductance of nearly 1 G_0 [4]. The corresponding conductance plateaus in breaking traces show statistically preferred lengths, which can be related to an integer

number of atoms in the chains [5]. We have carried out an analysis of the plateau lengths in the interval from 0.5 to 1.2 G_0. Figure S1 presents a histogram of the conductance plateau length in micrometers of bending. The two peaks just above 4 and 8 μm indicate the formation of chains of gold atoms and can be used to calibrate the displacement ratio. Earlier experiments have established that the interatomic distance in these chains is 2.5 Å [5]. This allows us to estimate the displacement ratio, which is defined as $\Delta d/\Delta w$, where d is the electrode displacement, and w the change in motor position. This yields a displacement ratio of $6\cdot10^{-5}$. A similar value is obtained by analyzing the exponential decrease of the tunneling conductance with increasing substrate deflection [6].

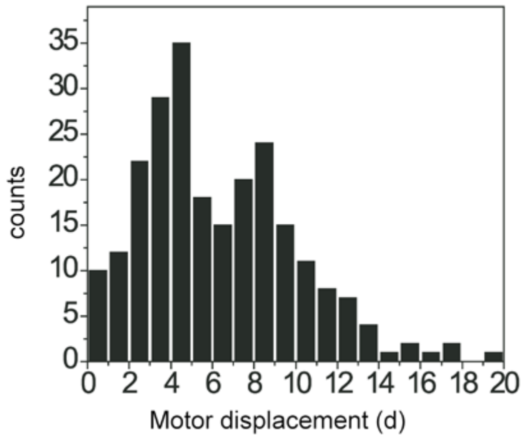

Figure S1: Histogram of the motor displacement in the conductance interval 0.5–1.2 G_0 used for the calibration of the displacement ratio of a clean gold MCBJ at 11 K. The data were obtained during 240 individual breaking and fusing cycles at a bias of 50 mV. The double peak structure indicates the formation of monatomic chains.

References

1. Adler, A. D.; Longo, F. R.; Finarelli, J. D.; Goldmacher, J.; Assour, J.; Korsakoff, L. *J. Org. Chem.* **1967,** *32,* 476. doi:10.1021/jo01288a053
2. Grozema, F. C.; Houarner-Rassin, C.; Prins, P.; Siebbeles, L. D. A.; Anderson, H. L. *J. Am. Chem. Soc.* **2007,** *129,* 13370. doi:10.1021/ja0751274
3. Martin, C. A.; Ding, D.; Sørensen, J. K.; Bjørnholm, T.; van Ruitenbeek, J. M.; van der Zant, H. S. J. *J. Am. Chem. Soc.* **2008,** *130,* 13198. doi:10.1021/ja804699a
4. Yanson, A. I.; Bollinger, G. R.; van den Brom, H. E.; Agraït, N.; van Ruitenbeek, J. M. *Nature* **1998,** *395,* 783. doi:10.1038/27405
5. Untiedt, C.; Yanson, A. I.; Grande, R.; Rubio-Bollinger, G.; Agraït, N.; Vieira, S.; van Ruitenbeek, J. M. *Phys. Rev. B* **2002,** *66,* 085418. doi:10.1103/PhysRevB.66.085418
6. Martin, C. A. Charge transport through single molecules in two- and three-terminal mechanical break junctions. Ph.D. Thesis, Delft University of Technology, The Netherlands, 2010.
